# Effects of Protective Mechanical Ventilation With Different PEEP Levels on Alveolar Damage and Inflammation in a Model of Open Abdominal Surgery: A Randomized Study in Obese Versus Non-obese Rats

**DOI:** 10.3389/fphys.2019.01513

**Published:** 2019-12-17

**Authors:** Lígia de A. Maia, Marcos V. S. Fernandes, Raquel S. Santos, Laís C. Agra, Anna Carolinna Carvalho, Nazareth de N. Rocha, Milena V. Oliveira, Cíntia L. Santos, Marcelo M. Morales, Vera L. Capelozzi, Sergio A. L. Souza, Bianca Gutfilen, Marcus J. Schultz, Marcelo Gama de Abreu, Paolo Pelosi, Pedro L. Silva, Patricia R. M. Rocco

**Affiliations:** ^1^Laboratory of Pulmonary Investigation, Carlos Chagas Filho Biophysics Institute, Federal University of Rio de Janeiro, Rio de Janeiro, Brazil; ^2^Department of Physiology and Pharmacology, Biomedical Institute, Fluminense Federal University, Niterói, Brazil; ^3^Laboratory of Cellular and Molecular Physiology, Carlos Chagas Filho Biophysics Institute, Federal University of Rio de Janeiro, Rio de Janeiro, Brazil; ^4^Department of Pathology, School of Medicine, University of São Paulo, São Paulo, Brazil; ^5^Nuclear Medicine Service, Clementino Fraga Filho University Hospital, Federal University of Rio de Janeiro, Rio de Janeiro, Brazil; ^6^Department of Intensive Care, Laboratory of Experimental Intensive Care and Anesthesiology (L.E.I.C.A.), Amsterdam University Medical Centers, University of Amsterdam, Amsterdam, Netherlands; ^7^Department of Anesthesiology and Intensive Care Therapy, Pulmonary Engineering Group, University Hospital Carl Gustav Carus, Dresden University of Technology, Dresden, Germany; ^8^Department of Surgical Sciences and Integrated Diagnostics, University of Genoa, Genoa, Italy; ^9^San Martino Policlinico Hospital, IRCCS for Oncology and Neurosciences, Genoa, Italy

**Keywords:** inflammation, epithelial cell damage, mechanical ventilation, positive-end expiratory pressure, obesity

## Abstract

Intraoperative positive end-expiratory pressure (PEEP) has been proposed to restore lung volumes and improve respiratory function in obesity. However, the biological impact of different PEEP levels on the lungs in obesity remains unknown. We aimed to compare the effects of PEEP = 2 cmH_2_O versus PEEP = 6 cmH_2_O during ventilation with low tidal volumes on lung function, histology, and biological markers in obese and non-obese rats undergoing open abdominal surgery. Forty-two Wistar rats (21 obese, 21 non-obese) were anesthetized and tracheotomized, and laparotomy was performed with standardized bowel manipulation. Rats were randomly ventilated with protective tidal volume (7 ml/kg) at PEEP = 2 cmH_2_O or PEEP = 6 cmH_2_O for 4 h, after which they were euthanized. Lung mechanics and histology, alveolar epithelial cell integrity, and biological markers associated with pulmonary inflammation, alveolar stretch, extracellular matrix, and epithelial and endothelial cell damage were analyzed. In obese rats, PEEP = 6 cmH_2_O compared with PEEP = 2 cmH_2_O was associated with less alveolar collapse (*p* = 0.02). E-cadherin expression was not different between the two PEEP groups. Gene expressions of interleukin (IL)-6 (*p* = 0.01) and type III procollagen (*p* = 0.004), as well as protein levels of tumor necrosis factor-alpha (*p* = 0.016), were lower at PEEP = 6 cmH_2_O than at PEEP = 2 cmH_2_O. In non-obese animals, PEEP = 6 cmH_2_O compared with PEEP = 2 cmH_2_O led to increased hyperinflation, reduced e-cadherin (*p* = 0.04), and increased gene expression of IL-6 (*p* = 0.004) and protein levels of tumor necrosis factor-alpha (*p*-0.029), but no changes in fibrogenesis. In conclusion, PEEP = 6 cmH_2_O reduced lung damage and inflammation in an experimental model of mechanical ventilation for open abdominal surgery, but only in obese animals.

## Introduction

Several intraoperative ventilator strategies may prevent lung damage. Randomized clinical trials of intraoperative ventilation for abdominal surgery (Futier et al., 2013; Hemmes et al., 2014; Ferrando et al., 2018) have compared diverse ventilation strategies with respect to development of postoperative pulmonary complications (PPCs). In the IMPROVE trial (Futier et al., 2013), low tidal volume (V_T_) with moderate positive end-expiratory pressure (PEEP) and recruitment maneuvers (RMs) resulted in fewer PPCs compared with high-V_T_ and no PEEP. In the PROVHILO trial (Hemmes et al., 2014), low-V_T_ with high-PEEP levels and RMs, compared with low-V_T_ and low-PEEP without RMs, did not protect against PPCs. In the iPROVE trial (Ferrando et al., 2018), higher PEEP or individualized PEEP setting compared with lower PEEP did not result in fewer PPCs. In an animal model of open abdominal surgery, both low-V_T_ and moderate to high-PEEP and RMs resulted in lower driving pressure, mechanical power, and lung damage (Maia et al., 2017). The PROBESE trial (Bluth et al., 2019) compared high-PEEP and RM versus low-PEEP at low V_T_ and found no significant differences in PPCs.

Obesity is a growing problem worldwide, which means that an increasing number of surgeries are being performed in obese patients (Schumann et al., 2015). Obese patients undergoing anesthesia have reduced lung volumes (Silva et al., 2012), which can be exacerbated by low V_T_, increasing atelectasis (Goldenberg et al., 2014). Intraoperative PEEP has been proposed to restore lung volumes and improve respiratory function (Reinius et al., 2009; Hedenstierna and Edmark, 2010; Imber et al., 2016). However, the biological impact of different levels of PEEP on the lungs in obesity remains unknown. We hypothesized that a protective tidal volume with a PEEP of 6 cmH_2_O (high PEEP for rats) might result in less mechanical and biological stress compared to a PEEP of 2 cmH_2_O (low PEEP for rats) during open abdominal surgery under general anesthesia in obese rats. The present study aimed to evaluate the impact of mechanical ventilation with high and low PEEP, both under low V_T_, on lung mechanics and histology, alveolar epithelial cell integrity, and biological markers associated with pulmonary inflammation, alveolar stretch, extracellular matrix, and epithelial and endothelial cell damage during open abdominal surgery in non-obese and obese rats.

## Materials and Methods

### Ethics Statement

This study was approved by the Research Ethics Committee of the Federal University of Rio de Janeiro Health Sciences Center (CEUA-117/16), Rio de Janeiro, Brazil (chair: Prof. Marcel Frajblat). All animals received humane care in compliance with the “Principles of Laboratory Animal Care” formulated by the National Society for Medical Research and the *Guide for the Care and Use of Laboratory Animals* prepared by the U.S. National Academy of Sciences. Experiments conformed with the “European Convention for the Protection of Vertebrate Animals used for Experimental and other Scientific Purposes” (Council of Europe No 123, Strasbourg 1985), and the present study followed the ARRIVE guidelines for reporting of animal research (Kilkenny et al., 2010).

### Animal Preparation and Experimental Protocol

Forty-two Wistar rats were kept in a temperature-controlled room (23–24°C) with artificial dark–light cycles (lights on at 7 am. and off at 7 pm.). Virgin female rats (3 months old) were caged with male rats at a proportion of 3:1. During pregnancy and lactation, each female was housed in an individual cage with free access to water and food (commercial rat chow). To induce postnatal obesity (Ob group), 3 days after birth, litters were culled to three males per litter. In non-obese animals (nonOb group), the litter size was adjusted to 10 pups per litter. After weaning (day 21), both nonOb and Ob animals received commercial diet. From postnatal days 21 to 180, offspring body weight (g) was monitored every 7 days. At postnatal day 150, chest computed tomography was performed in nonOb (*n* = 14) and Ob animals (*n* = 14) to characterize obesity (see Supplementary Figure S1, Supplementary Digital Content S1, which describes additional methods).

At postnatal day 180, nonOb (*n* = 21) and Ob (*n* = 21) animals were sedated (diazepam 10 mg/kg intraperitoneally) and anesthetized (ketamine 75 mg/kg and midazolam 2 mg/kg intraperitoneally). The tail vein was cannulated (Jelco 24G, Becton, Dickinson and Company, New Jersey, United States) for continuous infusion of 50 mg.kg^–1^.h^–1^ ketamine, 2 mg.kg^–1^.h^–1^ midazolam, and 7 mL.kg^–1^.h^–1^ Ringer’s lactate (B. Braun, Rio de Janeiro, Brazil) during mechanical ventilation. Gelafundin^®^ 4% (B. Braun, São Gonçalo, RJ, Brazil) was administered intravenously (in 0.5-mL increments) as needed to maintain mean arterial pressure (MAP) >60 mmHg. Depth of anesthesia was evaluated by the response to light touch with a fingertip on the rat’s whiskers (0 = awake, fully responsive to surroundings; 1 = not responsive to surroundings, rapid response to whisker stimulation; 2 = slow response; 3 = unresponsive to whisker stimulation), pupil diameter, position of the nictitating membrane, and movement in response to tail stimulation (Heil et al., 2016). Experiments were started when responses to a noise stimulus (handclap), whisker stimulation, and tail clamping were absent.

Local anesthetic (2% lidocaine, 0.4 mL) was infiltrated and a tracheostomy was performed via a midline neck incision for a 14-gauge cannula.

A catheter (18G, Arrow International, United States) was placed in the right internal carotid artery for blood sampling and gas analysis (Radiometer ABL80 FLEX, Copenhagen, NV, Denmark), as well as monitoring of MAP. Heart rate (HR), MAP, and rectal temperature were continuously monitored (Networked Multiparameter Veterinary Monitor LifeWindow 6000V, Digicare Animal Health, Boynton Beach, FL, United States). Body temperature was maintained at 37.5 ± 1°C using a heating bed.

Animals were then paralyzed (pancuronium 0.4 mg intramuscularly, followed by 1 mg/kg/h intravenously) (Spieth et al., 2015) and mechanically ventilated (Servo-I; MAQUET, Solna, Sweden) in volume-controlled mode with V_T_ = 7 mL/kg, RR to maintain normocapnia (PaCO_2_ = 35–45 mmHg; around 45 bpm), inspiratory-to-expiratory ratio = 1:2, fraction of inspired oxygen = 0.4, and PEEP = 2 cmH_2_O for 5 min (Baseline). Arterial blood (300 μL) was drawn into a heparinized syringe to determine arterial oxygen partial pressure (PaO_2_), arterial carbon dioxide partial pressure (PaCO_2_), and arterial pH (pHa; ABL80 FLEX, Radiometer, Copenhagen, Denmark). Blood gas analysis was also performed 10 min after laparotomy and at the end of the experiments. FiO_2_ was maintained at 0.4 throughout the experiments. Obese and non-obese rats were then randomly assigned, using closed sealed envelopes, to receive the same protective V_T_ (7 mL/kg) and two different PEEP levels: 2 cmH_2_O (PEEP2) and 6 cmH_2_O (PEEP6).

After group allocation (*n* = 7/group), laparotomy was performed, and animals were ventilated in volume-controlled mode for 4 h. The respiratory rate (RR) was adjusted to reach a minute ventilation of 160 mL/min. At the start of mechanical ventilation and every hour thereafter, a standardized bowel manipulation was performed as follows: under sterile conditions, lateral retractors were carefully placed, the bowel was gently taken out of the abdominal cavity and reintroduced within 20 s. The retractors were left in place, and the abdominal cavity was continuously humidified with warmed saline at 37°C. Lung mechanics, heart rate, MAP, and the amount of fluids infused were measured hourly. At the end of the experiment, animals were killed by injection of sodium thiopental (60 mg/kg), the lungs were extracted for postmortem analysis, and visceral fat mass was excised (mesenteric, epididymal and retroperitoneal white adipose tissue) and immediately weighed for evaluation of central adiposity. Seven nonOb and Ob rats were not tracheotomized, operated, or mechanically ventilated, and constituted the healthy, non-operated and non-ventilated control groups (NV-nonOb and NV-Ob, respectively).

### Respiratory Data Acquisition and Processing

Airflow, as well as airway (Paw) and esophageal (Pes) pressures, were continuously measured. A pneumotachograph (internal diameter = 1.5 mm, length = 4.2 cm, distance between side ports = 2.1 cm) was connected to the tracheal cannula for airflow (V’) measurements (Mortola and Noworaj, 1983). The pressure gradient across the pneumotachograph was determined using a SCIREQ differential pressure transducer (UT-PDP-02, SCIREQ, Montreal, QC, Canada). V_T_ was calculated by digital integration of the airflow signal obtained from the pneumotachograph, connected to the Y-piece of the ventilator tubing, while RR was calculated from the Pes swings as the frequency per minute of each type of breathing cycle. Airway pressure was measured with a SCIREQ differential pressure transducer (UT-PDP-75, SCIREQ, Montreal, QC, Canada). Esophageal pressure was measured using a 30-cm-long water-filled catheter (PE-205, Becton, Dickinson and Company) with side holes at the tip, connected to a differential pressure transducer (UT-PL-400, SCIREQ, Montreal, QC, Canada). The catheter was passed into the stomach and then slowly returned into the esophagus; its proper positioning was assessed with the “occlusion test”, as described elsewhere (Samary et al., 2015). Transpulmonary pressure (PL) was calculated during inspiration and expiration as the difference between Paw and Pes (Samary et al., 2015; Spieth et al., 2015; Felix et al., 2019). Values were recorded continuously using LabView-based software (National Instruments, Austin, TX). All signals were filtered (100 Hz), amplified in a 4-channel conditioner (SC-24; SCIREQ), and sampled at 200 Hz with a 12-bit analog-to-digital converter (National Instruments) (Felix et al., 2019).

Lung mechanics were assessed every hour by occluding the airway at end-inspiration for 5 s (Samary et al., 2015). Static lung elastance (Est,L = (P,L_end–insp_-P,L_end–exp_)/V_T_) and chest wall elastance (Est,w = (P,es_end–insp_-Pes,_end–exp_)/V_T_) were calculated [P,L_end–insp =_ P,rs_end–insp_ (Paw at end inspiratory occlusion) – P,es_end–insp_ (Pes at end-inspiratory occlusion) and P,L_end–exp_ = PEEP-Pes,_end–exp_ (Pes at end-expiration)] offline using a routine written in MATLAB (version R2007a; The Mathworks Inc., Natick, MA, United States). All analyses were performed in a blinded manner, i.e., the observer was unaware of the experimental protocol.

### Histology

Immediately after euthanasia, heparin (1000 IU) was injected into the tail vein, the trachea was clamped at end-expiration, and the lungs were removed *en bloc* at PEEP = 3 cm H_2_O in all groups. The left lung was frozen in liquid nitrogen and submerged in Carnoy’s solution (Pássaro et al., 2009; Felix et al., 2019). Slices (4 μm thick) were stained with hematoxylin and eosin. Lung morphometric analysis was performed using an integrating eyepiece with a coherent system consisting of a grid with 100 points and 50 lines of known length coupled to a conventional light microscope (Olympus BX51, Olympus Latin America Inc., São Paulo, Brazil). The volume fractions of the lung occupied by collapsed alveoli (alveoli with rough or plicate walls) and hyperinflated structures (alveolar ducts, alveolar sacs, or alveoli wider than 120 μm) were determined by the point-counting technique at a magnification of × 200 across 10 random, non-coincident microscopic fields (Hsia et al., 2010; Wierzchon et al., 2017).

### Immunohistochemistry

The right lower lung was immersed in immunohistochemistry solution. To evaluate the degree of epithelial cell damage, expression of E-cadherin (the major transmembrane protein of the adherens junction) was analyzed (Maia et al., 2017). Immunohistochemical procedures were performed on lung sections using a mouse polyclonal antibody against E-cadherin (cat. 610181, BD Transduction Laboratories, 1:300). Visualization and image capture were performed using a light microscope (Eclipse E800, Nikon, Japan) coupled to a digital camera (Evolution, Media Cybernetics Inc., Rockville, MD, United States) and Q-Capture 2.95.0 graphic interface software (version 2.0.5; Quantitative Imaging, Surrey, British Columbia, Canada). Expression of E-cadherin was analyzed using ImagePro Plus software (version 4.5.1, Media Cybernetics). The pathologist or technician working on lung morphometry and immunohistochemistry was blinded to the nature of the study.

### Molecular Biology

The right middle lung was flash-frozen by immersion in liquid nitrogen and stored at −80°C for quantification of mRNA expression. Quantitative real-time reverse transcription polymerase chain reaction was performed to measure markers associated with inflammation [interleukin (IL)-6], mechanical pulmonary stretch (amphiregulin), epithelial cell injury [club cell secretory protein (CC-16)], endothelial cell damage [vascular cell adhesion molecule (VCAM)-1], and extracellular matrix damage [type III procollagen (PCIII), decorin, and metalloproteinase-9 (MMP-9)] (see Supplementary Table S1, Supplementary Digital Content S1, for primers). Central slices of the right lung were cut, collected in cryotubes, flash-frozen by immersion in liquid nitrogen, and stored at −80°C. Total RNA was extracted from frozen tissues using the RNeasy Plus Mini Kit (Qiagen, Hilden, Germany), following the manufacturer’s recommendations. RNA concentrations were measured by spectrophotometry in a Nanodrop ND-1000 system (Thermo Scientific, Wilmington, DE, United States). First-strand cDNA was synthesized from total RNA using a Quantitec reverse transcription kit (Qiagen, Hilden, Germany). Relative mRNA levels were measured by SYBR green detection in an ABI 7500 real-time PCR system (Applied Biosystems, Foster City, CA, United States). Samples were run in triplicate. For each sample, the expression of each gene was normalized to the acidic ribosomal phosphoprotein P0 (*36B4*) housekeeping gene and expressed as fold change relative to respective NV-nonOb (*n* = 7) and NV-Ob (*n* = 7) animals, using the 2^–ΔΔ*Ct*^ method, where ΔCt = Ct (target gene)– Ct (reference gene) (Schmittgen and Livak, 2008).

### Enzyme-Linked Immunosorbent Assay (ELISA)

The right upper lung was immediately frozen in liquid nitrogen and stored at −80°C for ELISA. Tumor necrosis factor (TNF)-α levels were quantified by ELISA in the lung tissue homogenate. All procedures were done according to the manufacturer’s protocol (PeproTech, London, United Kingdom) and normalized to total protein as assessed by Bradford’s reagent (Sigma-Aldrich, St Louis, MO, United States).

### Statistical Analysis

The sample size calculation of each group was based on our experimental experience, which allowed detection of significant differences with the smallest possible number of animals, and on the respiratory effects observed in a previous study in rodents using comparable ventilator settings. A sample size of 7 animals per group would provide the appropriate power (1 − β = 0.8) to identify significant (α = 0.05) differences in IL-6 gene expression in lung tissue between ventilatory strategies based on PEEP2 and PEEP6 in obese animals (primary outcome), taking into account an effect size *d* = 1.76, a 2-sided test, and a sample size ratio = 1 (G^∗^Power 3.1.9.2, University of Düsseldorf, Düsseldorf, Germany). The secondary outcomes were lung mechanics and histology, alveolar epithelial integrity, and pulmonary inflammation.

Variables were tested for normality using the Kolmogorov–Smirnov test. Parametric data were expressed as means ±SD, and non-parametric data as median (interquartile range). To compare lung mechanics and blood gas analysis over time, two-way repeated-measures ANOVA followed by the Bonferroni test was used. Fraction area of alveolar collapse and hyperinflation, E-cadherin expression, and the protein levels of TNF-α in lung tissue between nonOb and Ob groups ventilated with PEEP6 or PEEP2 were compared using two-way ANOVA followed by Bonferroni’s test. Obese and non-obese non-ventilated animals presented different biological behavior; thus, the expression of biological markers in the Ob and nonOb groups ventilated with PEEP6 or PEEP2 was compared separately using the Mann–Whitney *U* test. All tests were performed in GraphPad Prism version 6.07 (GraphPad Software, San Diego, CA, United States). The significance level was set at 5%.

## Results

A characterization of the rat model of obesity used in this study is presented in Supplementary Digital Content S2 (see Supplementary Figures S2–S4, which describe the comparisons between nonOb and Ob animals). The presentation of respiratory parameters and mean arterial pressure of nonOb and Ob animals at Baseline, are presented in Supplementary Digital Content S2 (Supplementary Table S2). Est,L and mean arterial pressure were higher and PaO_2_/FiO_2_ were lower in Ob than in nonOb animals, regardless of PEEP levels. No significant differences were observed in the amount of fluids between groups: mean ±SD, nonOb/PEEP2 = 19 ± 5 mL; nonOb/PEEP6 = 15 ± 2 mL; Ob/PEEP2 = 20 ± 4 mL; Ob/PEEP6 = 13 ± 7 mL.

### Effects of PEEP in Obese and Non-obese Rats

#### Obese Rats

**FIGURE 1:**
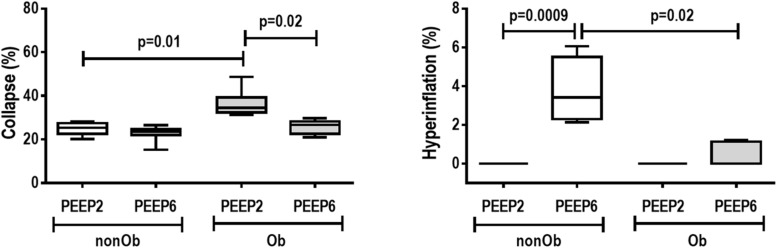
Lung morphometry in mechanically ventilated non-obese (nonOb) and obese (Ob) groups: Low-PEEP (2 cmH_2_O) and High-PEEP (6 cmH_2_O). Fraction area of alveolar collapse and lung hyperinflation. Boxes show the interquartile range (25th–75th percentile), while whiskers encompass the range (minimum-maximum) and horizontal lines represent the median in seven animals/group.

**FIGURE 2:**
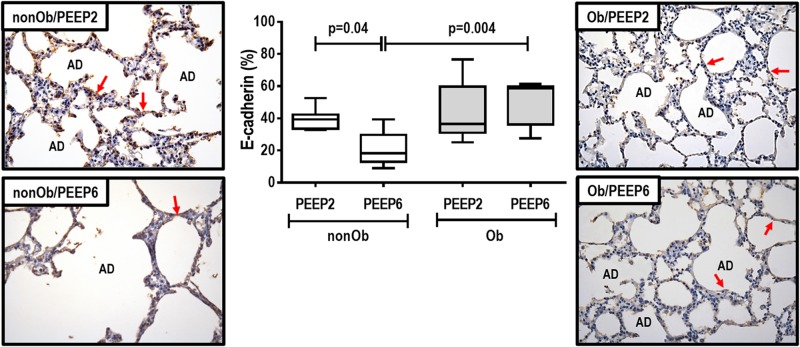
E-cadherin tissue expression and representative photomicrographs of immunohistochemical staining for E-cadherin in mechanically ventilated obese groups: Low-PEEP (2 cmH_2_O) and High-PEEP (6 cmH_2_O). Boxes show the interquartile range (25th–75th percentile), while whiskers encompass the range (minimum-maximum) and horizontal lines represent the median in seven animals/group. Red arrows represent E-cadherin immunohistochemical staining (brown). AD, alveolar ducts. Original magnification × 400.

**FIGURE 3:**
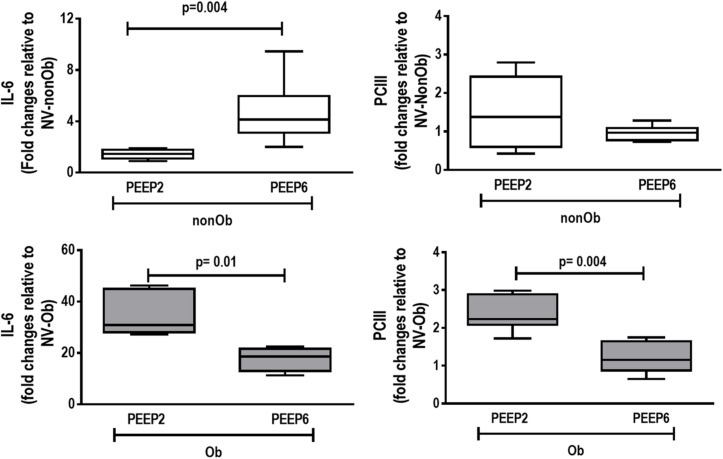
Expression of biologic markers associated with lung damage in non-obese (nonOb) and obese (Ob) mechanically ventilated groups. Low-PEEP (2 cmH_2_O) and High-PEEP (6 cmH_2_O). Real-time polymerase chain reaction analysis of interleukin (IL)-6 and type III procollagen (PCIII). Relative gene expression was calculated as a ratio of average expression of each gene to the reference gene (*36B4*) and expressed as fold change relative to non-ventilated animals (NV). Boxes show the interquartile range (25th–75th percentile), while whiskers encompass the range (minimum-maximum) and horizontal lines represent the median in seven animals/group.

**FIGURE 4:**
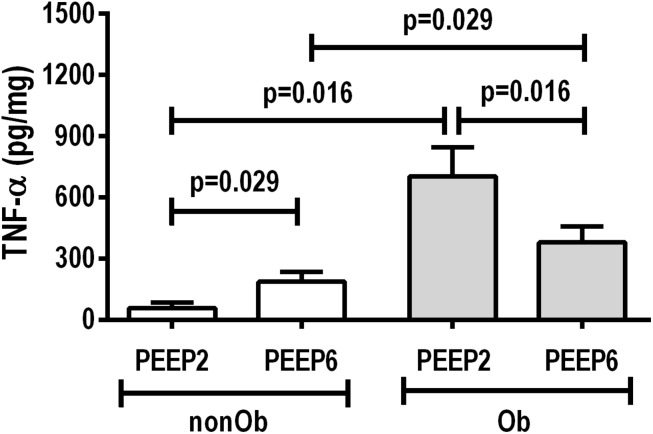
Protein levels of tumor necrosis factor (TNF)-α in lung tissue homogenate in non-obese (nonOb) and obese (Ob) mechanically ventilated groups: Low-PEEP (2 cmH_2_O) and High-PEEP (6 cmH_2_O). Bars represent means +SD of seven animals in each group.

At END, PaO_2_/FiO_2_ was higher in Ob/PEEP6 than Ob/PEEP2, with no significant differences in pHa and PaCO_2_ (Table 1). Est,L and Est,w did not differ significantly between PEEP levels (Table 2). Alveolar collapse was lower in the Ob/PEEP6 group (Figure 1). No difference was observed in E-cadherin expression (Figure 2). Amphiregulin, CC-16, VCAM-1, decorin, and MMP-9 gene expressions in lung tissue did not differ between the Ob/PEEP6 and Ob/PEEP2 groups (Supplementary Digital Content S2, Supplementary Figure S5). On the other hand, IL-6 and PCIII gene expressions (Figure 3) and protein levels of TNF-α (Figure 4) were lower in Ob/PEEP6 compared with Ob/PEEP2 animals.

**TABLE 1:** Arterial blood gases.

**Parameter**	**Time point**	**NonOb**		**Ob**		**Time effect**	**Group effect**	**Interaction**
		**PEEP2**	**PEEP6**	**PEEP2**	**PEEP6**			
pHa	INITIAL	7.34 ± 0.06	7.35 ± 0.04	7.37 ± 0.04	7.37 ± 0.05	*P* = 0.950	*P* = 0.264	*P* = 0.785
	END	7.34 ± 0.03	7.36 ± 0.03	7.38 ± 0.05	7.35 ± 0.03			
PaCO_2_ (mmHg)	INITIAL	41 ± 4	40 ± 3	39 ± 4	41 ± 3	*P* = 0.8327	*P* = 0.1423	*P* = 0.576
	END	42 ± 3	41 ± 3	39 ± 3	39 ± 2			
PaO_2_/FiO_2_	INITIAL	318 ± 53	374 ± 43	243 ± 67	329 ± 26	*P* = 0.885	*P* < 0.0001	*P* = 0.2084
	END	283 ± 87	395 ± 39^†^	222 ± 61	372 ± 24†			

*Blood gas analysis 10 min after laparotomy (INITIAL) and at the end of 4 h of mechanical ventilation (END). Values are mean ± SD of seven rats in each group. nonOb, non-obese; nonOb/PEEP2, V_*T*_ = 7 mL/kg with PEEP = 2 cmH_2_O; nonOb/PEEP6, V_*T*_ = 7 mL/kg with PEEP = 6cmH_2_O; Ob, obese; Ob/PEEP2, V_*T*_ = 7 mL/kg with PEEP = 2 cmH_2_O; Ob/PEEP6, V_*T*_ = 7 mL/kg with PEEP = 6 cmH_2_O. Two-way repeated-measures ANOVA followed by the Bonferroni test. ^†^*p* < 0.05 vs. nonOb/PEEP2-END; ‡*p* < 0.05 vs. Ob/PEEP2-END.*

**TABLE 2:** Respiratory parameters.

**Parameter**	**Time point**	**NonOb**		**Ob**		**Time effect**	**Group effect**	**Interaction**
		**PEEP2**	**PEEP6**	**PEEP2**	**PEEP6**			
V_T_ (mL/kg)	INITIAL	6.8 ± 0.5	6.8 ± 0.4	6.7 ± 0.6	6.8 ± 0.5	*P* = 0.395	*P* = 0.461	*P* = 0.881
	END	6.7 ± 0.7	7.0 ± 0.4	6.8 ± 0.7	6.7 ± 0.4			
RR (bpm)	INITIAL	45 ± 3	44 ± 2	43 ± 3	42 ± 2	*P* = 0.790	*P* = 0.090	*P* = 0.932
	END	45 ± 4	44 ± 2	44 ± 2	44 ± 2			
Est,L (cmH_2_O/mL)	INITIAL	2.2 ± 0.3	2.2 ± 0.3	3.1 ± 0.6^∗^	3.0 ± 0.4^∗∗^	*P* < 0.0001	*P* < 0.0001	*P* = 0.1788
	END	3.0 ± 0.2^∗^	2.8 ± 0.1	3.6 ± 0.4^†^	3.1 ± 0.5			
Est,w (cmH_2_O/mL)	INITIAL	0.37 ± 0.04	0.39 ± 0.05	0.40 ± 0.04	0.40 ± 0.05	*P* = 0.822	*P* = 0.541	*P* = 0.667
	END	0.39 ± 0.03	0.37 ± 0.05	0.40 ± 0.03	0.40 ± 0.04			

*Respiratory parameters 10 min after laparotomy (INITIAL) and after 4 h of mechanical ventilation (END). Values are mean ± SD of seven rats in each group. nonOb, non-obese; nonOb/PEEP2, V_*T*_ = 7 mL/kg with PEEP = 2 cmH_2_O; nonOb/PEEP6, V_*T*_ = 7 mL/kg with PEEP = 6 cmH_2_O; Ob, obese; Ob/PEEP2, V_*T*_ = 7 mL/kg with PEEP = 2 cmH_2_O; Ob/PEEP6, V_*T*_ = 7 mL/kg with PEEP = 6 cmH_2_O; V_*T*_, tidal volume; RR, respiratory rate; Est,L, lung static elastance; Est,w, chest wall static elastance. ^∗^*p* < 0.05 vs. nonOb/PEEP2-INITIAL; ^∗∗^*p* < 0.05 vs. nonOb/PEEP6-INITIAL; *^†^p* < 0.05 vs. nonOb/PEEP2-END.*

#### Non-obese Rats

At END, PaO_2_/FiO_2_ was higher in nonOb/PEEP6 than in nonOb/PEEP2, with no significant differences in pHa and PaCO_2_ (Table 1). At INITIAL and END, Est,L and Est,w did not differ significantly between PEEP levels (Table 2), but was higher at END than INITIAL in the non-Ob/PEEP6 group (Table 2). The fraction area of alveolar collapse did not differ, and hyperinflation increased in the nonOb/PEEP6 group (Figure 1). E-cadherin expression was reduced in nonOb/PEEP6, suggesting alveolar epithelial cell damage (Figure 2). Gene expression of IL-6 was higher in nonOb/PEEP6 than nonOb/PEEP2 (Figure 3). No significant differences were observed in gene expressions of PCIII (Figure 3), amphiregulin, CC-16, VCAM-1, decorin, or MMP-9 (Supplementary Digital Content S2, Supplementary Figure S5). Protein levels of TNF-α were higher in nonOb/PEEP6 than nonOb/PEEP2 animals (Figure 4).

#### Obese Versus Non-obese Rats

At the same PEEP level (i.e., 2 or 6 cmH_2_O), pHa, PaCO_2_, and PaO_2_/FiO_2_ did not differ significantly between Ob and nonOb groups (Table 1). At INITIAL, Est,L was higher in Ob than nonOb animals regardless of PEEP level. At END, Est,L was higher in Ob than nonOb animals at PEEP2 (Table 2). At PEEP2, Ob animals exhibited greater alveolar collapse than their nonOb counterparts. At PEEP6, lung hyperinflation was lower (Figure 1) and E-cadherin expression higher (Figure 2) in Ob than in nonOb groups. TNF-α levels were also greater in Ob compared with nonOb animals, regardless of PEEP level (Figure 4).

## Discussion

In an experimental model of ventilation for open abdominal surgery, PEEP = 6 cmH_2_O compared to PEEP = 2 cmH_2_O resulted in (1) less alveolar collapse and a lower pro-inflammatory and fibrogenic response in obese rats; and (2) increased hyperinflation, epithelial cell damage, and lung inflammation in non-obese rats. Our data suggest that PEEP6 reduced lung inflammation and fibrosis in obese rats undergoing open abdominal surgery, whereas the same PEEP6 resulted in lung damage in non-obese animals.

In the present study, we used a well-established metabolic programing model of obesity, which, compared with other models based on dietary interventions (Heil et al., 2016; Maia et al., 2019), better resembles the major hallmarks of clinical obesity (Plagemann et al., 2009). The difference in body weight between obese and non-obese animals, although modest, was significant (*p* = 0.0006). In the present study, CT showed increased visceral fat mass, which may be associated with reduced pulmonary density, as well as higher heterogeneity and an increase in hypoaerated areas in the lung. These morphological changes resulted in increased Est,L and reduced oxygenation in obese compared to non-obese animals. A low V_T_ (6–8 mL/kg) was used since it has been associated with fewer PPCs (Severgnini et al., 2013). However, a recent study showed that obese patients are frequently ventilated with high-V_T_ and low-PEEP (Ball et al., 2018), a combination that may cause lung damage. Beyond V_T_, there are controversies regarding the level of PEEP to be used during open abdominal surgery in non-obese patients (Futier et al., 2013; Hemmes et al., 2014; Güldner et al., 2015; Ferrando et al., 2018). Adequate PEEP levels may reduce atelectasis and improve lung mechanics and oxygenation, while high-PEEP may yield overdistension and impair ventilation-perfusion ratio and hemodynamics (Pelosi and Gregoretti, 2010). The PEEP levels tested in the present study (2 and 6 cmH_2_O) represented a two-fold increase compared with humans (4 and 12 cmH_2_O), due to differences in transpulmonary pressures between humans and rats (Caironi et al., 2011). These PEEP levels were previously tested in clinical studies (Nestler et al., 2017; Pereira et al., 2018; Bluth et al., 2019).

To the best of our knowledge, this is the first standardized, randomized preclinical translational study to compare the functional, morphological, and biological impacts of two levels of PEEP (low and high) during open abdominal surgery in non-obese and obese rats.

In obese animals, lung mechanics did not differ between PEEP = 2 cmH_2_O and PEEP = 6 cmH_2_O. Oxygenation improved with PEEP = 6 cmH_2_O, which may be associated with reduced alveolar collapse and heterogeneity score, yielding a less pro-inflammatory response (as measured by TNF-α protein levels and IL-6 gene expression) and less fibrogenesis (as measured by PCIII gene expression) in lung tissue. The absence of lung hyperinflation may be explained by the lower end-expiratory lung volumes before the application of PEEP, and, probably, by the presence of more compliant alveoli. At PEEP = 2 cmH_2_O, the continuous opening and closing of collapsed alveolar units during tidal breath may increase shear stress, thus promoting lung injury, inflammation (Bilek et al., 2003), and fibrogenesis (Farias et al., 2005). The absence of hyperinflation was likely associated with the absence of changes in gene expression of amphiregulin (Dolinay et al., 2004).

In non-obese animals, PEEP = 6 cmH_2_O, compared to PEEP = 2 cmH_2_O, did not modify lung mechanical parameters, but oxygenation was higher at PEEP = 6 cmH_2_O due to lung hyperinflation and increased ventilation-perfusion ratio. Alveolar collapse did not differ between PEEP = 2 cmH_2_O and PEEP = 6 cmH_2_O. PEEP = 6 cmH_2_O, compared to PEEP = 2 cmH_2_O, led to decreased E-cadherin as well as increased IL-6 gene expression and TNF-α protein content in lung tissue, suggesting damage to alveolar epithelial cells and greater lung inflammation, respectively. Based on these data, lung hyperinflation seemed to be associated with increased lung inflammatory response when compared with atelectasis *per se*. This reinforces the hypothesis that volutrauma is more injurious than atelectrauma in non-obese rats. The absence of changes in mediators other than those associated with inflammation is consistent with *post hoc* analyses of the PROVHILO trial, in which high-PEEP leads to minimal changes in biological markers (Serpa Neto et al., 2017).

The fact that lung mechanics were not affected by PEEP in non-obese or obese animals at END may be attributed to the following mechanisms: (1) different volume-pressure curve slope and position, resulting in diverse lung mechanical properties; and (2) different regional mechanical behavior of alveoli (less or more compliant), which included those opened before PEEP = 6 cmH_2_O and those reopened after PEEP = 6 cmH_2_O. In addition, chest wall mechanical properties did not differ among groups or over time. This may be explained by the increased abdominal compliance of small animals, which may balance the effects of the open-abdomen preparation.

In obese compared to non-obese animals, at PEEP = 6 cmH_2_O, lung hyperinflation was reduced, whereas E-cadherin expression increased. This increase in E-cadherin expression suggests that the integrity of alveolar epithelial cells was preserved in obese animals (Kasper et al., 1995; Goto et al., 2000). At PEEP = 2 cmH_2_O, alveolar collapse was increased and TNF-α levels in lung tissue were higher in obese animals, probably due to the enhanced lung inflammatory response associated with obesity (Mancuso, 2010).

A multicenter, international randomized controlled trial (PROBESE) compared the effects of two different levels of intraoperative PEEP (4 cmH_2_O versus 12 cmH_2_O) during protective low-V_T_ ventilation in obese patients and observed no differences in PPCs (Bluth et al., 2019). Taking into account species differences, the PEEP levels used in our study and in PROBESE are comparable, but comparison between these two studies is unwarranted, since laparoscopy and recruitment maneuvers were performed in most patients.

### Limitations

This study has some limitations that need to be addressed. First, a postnatal early overnutrition model was used; therefore, our results cannot be extrapolated to other models, including genetic variation (Kordonowy et al., 2012) and diet-induced obesity (Heil et al., 2016). Second, even considering the shorter lifespan of rats, the time of exposure of our animals to changes induced by obesity was relatively short. Nevertheless, since rats have a higher metabolic basal rate, a period of 150 days in rats would correspond to 14 years in humans (West and West, 2013). Third, our model of open abdominal surgery (laparotomy plus bowel manipulation), despite widespread use for experimental research (Maia et al., 2017), does not reproduce all aspects of the complex clinical scenario, where surgical trauma may be accompanied by bleeding and hemodynamic impairment. Fourth, in order to properly compare lung morphometry among groups, all animals had their PEEP level adjusted to 3 cmH_2_O for 2 min. Fifth, according to clinical trials and observational studies, ventilator strategies which feature high-PEEP are usually followed by RMs (Reinius et al., 2009; Pirrone et al., 2016). We chose to not include RMs or PEEP titration in our study, since this would have increased the number of groups and might have decreased the statistical power to find differences among groups. Sixth, the end-expiratory lung volume was not measured. Thus, further studies are required to better understand the changes in regional lung mechanical changes after each PEEP level.

## Conclusion

In an experimental model of mechanical ventilation for open abdominal surgery, PEEP = 6 cmH_2_O reduced lung inflammation in obese rats. Conversely, in non-obese animals, PEEP = 6 cmH_2_O increased inflammation and alveolar epithelial cell damage.

## Data Availability Statement

The datasets generated for this study are available on request to the corresponding author.

## Author Contributions

LM and MF participated in the design of the study, carried out the experiments, performed the data analyses, and drafted the manuscript. RS, LA, AC, NR, and MO contributed to the study design and carried out the experiments. MF and VC performed the analyses of lung morphology. SS and BG performed the CT analyses. LM, RS, and AC performed the analyses of lung mechanics. CS and MM carried out the molecular biology analyses. MS, MG, PP, PS, and PR contributed to the study design, supervised the experimental work and statistical analysis, wrote the manuscript, and supervised the entire project. All authors read and approved the final manuscript.

## Conflict of Interest

The authors declare that the research was conducted in the absence of any commercial or financial relationships that could be construed as a potential conflict of interest. The reviewer CB declared a shared affiliation, with no collaboration, with one of the authors, VC, to the handling Editor at the time of review.
