# Supplementary material for: Effects of Protective Mechanical Ventilation With Different PEEP Levels on Alveolar Damage and Inflammation in a Model of Open Abdominal Surgery: A Randomized Study in Obese Versus Non-obese Rats
Source: Front Physiol. 2019 Dec 17;10:1513. doi: 10.3389/fphys.2019.01513 (PMC6930834; doi:10.3389/fphys.2019.01513)
Supplement: Supplementary file 1 [file Data_Sheet_1.docx]

**Supplemental Digital Content 1**

**Additional methods**

**Experimental model** **of postnatal early overfeeding**

To induce early overfeeding (EO) during lactation, 3 days after birth, litters were culled to three males each (Obese)^1,2^. For the non-Obese group, litters were culled to 10 pups per dam. Two male rats were randomly chosen from each of the 14 different litters (14 Obese, 14 non-Obese) for subsequent analysis. After postnatal day 21 (PN21), which corresponds to the end of the weaning period, both groups had free access to water and a standard diet. From PN21 until PN180, offspring body weight (g) was monitored every 7 days.

**Computed tomography scan**

At PN150, animals were anesthetized (75 mg/kg ketamine and 2 mg/kg midazolam intraperitoneally) and placed in dorsal recumbency for computed tomography (CT) of the chest. Lungs were scanned lengthwise in conventional mode using a fast-rotating GE PET/CT Optima 560 scanner (GE Healthcare, Boston, USA) with the following settings: 120 kV peak voltage, 80 mA tube current, and 16 ms exposure time. Contiguous slices 0.68 mm thick were obtained from the apex to the base of the lungs by incremental movement of the scanning table. Images were analyzed using a 100-mm field of view and a 512 × 512 reconstruction matrix in conventional mode. All slices were segmented and the CT number in Hounsfield units (HUs) used for classification as follows: −1024 HU to −900 UH, hyperaerated area; −900 HU to −500 HU, normally aerated area; −500 HU to −100 HU, hypoaerated area; and −100 HU to 100 HU, non-aerated area (Gattinoni et al., 2001). Lung mass was calculated by assuming each voxel was a linear combination of only air and tissue^3^.

For analysis of visceral adipose tissue, 0.68-mm axial slices were obtained without contrast. Sections of the caudal retroperitoneal region at the level of the lower pole of the kidneys were used, since this is the region where fatty tissue is observed, and the mesenteric fat appears sparse^4^.

**Experimental timeline**

At PN180, nonOb and Ob animals were sedated (10 mg/kg diazepam intraperitoneally), anesthetized (75 mg/kg ketamine and 2 mg/kg midazolam intraperitoneally), paralyzed (pancuronium 0.4 mg intramuscularly, followed by 1 mg/kg/h intravenously), and mechanically ventilated (Servo-I; MAQUET, Solna, Sweden) in volume-controlled mode for 4 hours according to the timeline below.

**
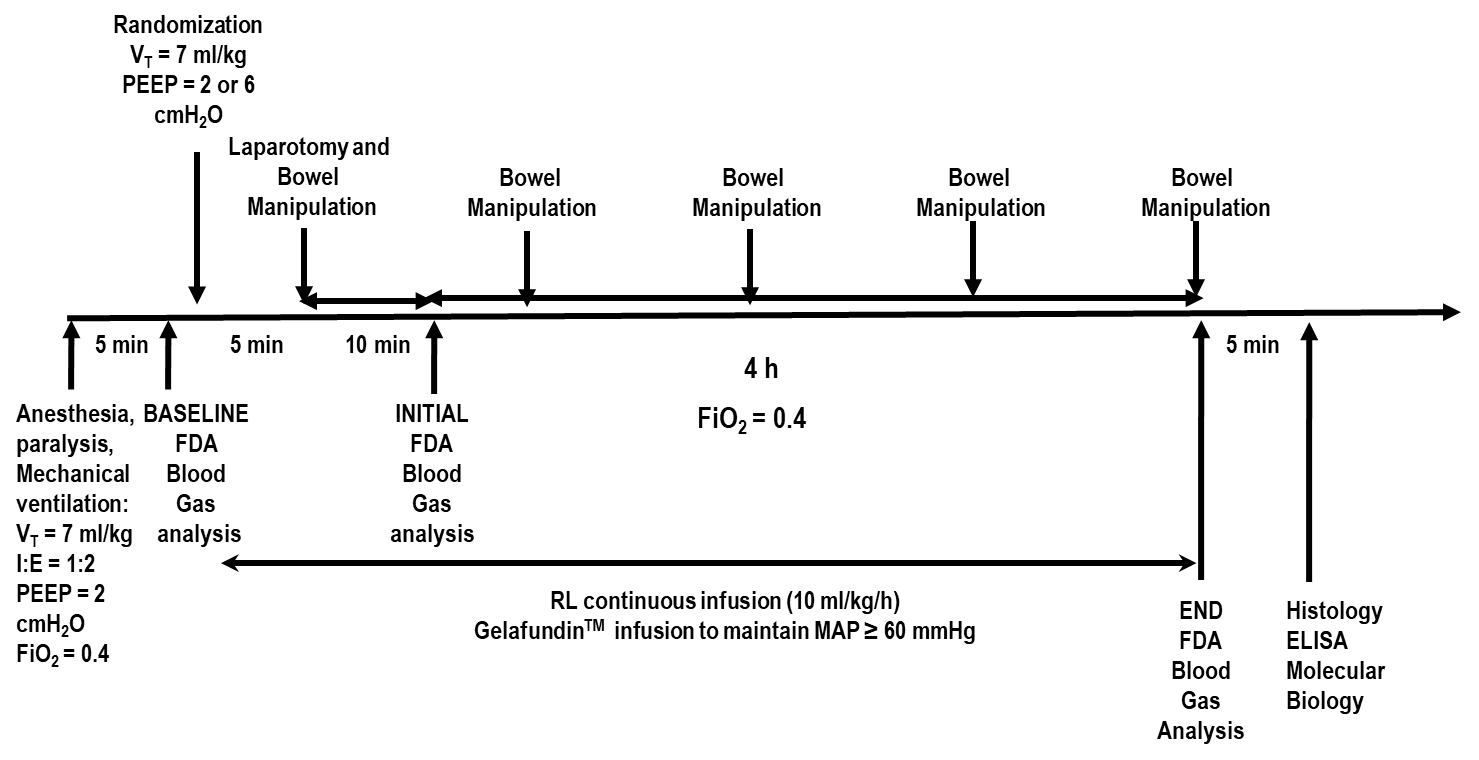
**

**Figure S1:** Timeline of experiments. V_T_, tidal volume; RR, respiratory rate; I:E, inspiratory-to-expiratory ratio; PEEP, positive end-expiratory pressure; FiO_2_, fraction of inspired oxygen; FDA, functional data acquisition; RL, Ringer’s lactate.

**Primers**

**Table S1.** Primers used for analysis of biological markers associated with inflammation,

alveolar pulmonary stretch, and damage to epithelial and endothelial cells and extracellular matrix.

| **Gene** | **Primer** | **Primer sequences (5′-3′)** |
| --- | --- | --- |
| IL-6 | Forward | CTC CGC AAG AGA CTT CCA G |
|  | Reverse | CTC CTC TCC GGA CTT GTG A |
| Amphiregulin | Forward | TTT CGC TGG CGC TCT CA |
|  | Reverse | TTC CAA CCC AGC TGC ATA ATG |
| CC-16 | Forward | GAT CG CCA TCA CAA TCA CTG |
|  | Reverse | GGT ATC CAC CAG CCT CTT CA |
| VCAM-1 | Forward | TGC ACG GTC CCT AAT GTG TA |
|  | Reverse | TGC CAA TTT CCT CCC TTA AA |
| PCIII | Forward | ACC TGG ACC ACA AGG ACA C |
|  | Reverse | TGG ACC CAT TTC ACC TTT C |
| Decorin | Forward | GAT CAG CCC AGA GGC ATT TA |
|  | Reverse | GCT CCA TTT TCA ATC CCA GA |
| MMP-9 | Forward | CCA CCG AGC TAT CCA CTC AT |
|  | Reverse | GTC CGG TTT CAG CAT GTT TT |
| *36B4* | Forward | AAT CCT GAG CGA TGT GCA G |
|  | Reverse | GCT GCC ATT GTC AAA CAC |

Forward and reverse oligonucleotide sequences of target gene primers: IL-6, interleukin-6; CC-16, club cell protein; VCAM-1, vascular cell adhesion molecule; PCIII, type III procollagen; MMP-9, metalloproteinase-9; *36B4*, acidic ribosomal phosphoprotein P0.

**REFERENCES**

1. Plagemann, A., Heidrich, I., Götz, F., Rohde, W., Dörner, G. (1992). Obesity and Enhanced Diabetes and Cardiovascular Risk in Adult Rats due to Early Postnatal Overfeeding. *Exp. Clin. Endocrinol* 99:154–158.
2. De Albuquerque Maia L, Lisboa P, de Oliveira, E., da Conceição, E.P., Lima, I.C., Lopes, R.T., et al. (2014) Bone structure and strength are enhanced in rats programmed by early overfeeding. *Hormone Metab Res.* 46:259–268.
3. Busse, N., Erwin, W., Pan, T. (2013). Evaluation of a semiautomated lung mass calculation technique for internal dosimetry applications. *Med. Phys.* 40:122503.
4. Raso, R., Paim, R., Pinheiro, S., Tavares, W.C. Júnior, Vasconcellos, L.S, Alberti, L. (2017). Effects of chronic consumption of green tea on weight and body fat distribution of Wistar rats evaluated by computed tomography. *Acta Cir Bras* 32:342–349.
