# Supplementary material for: Effects of Protective Mechanical Ventilation With Different PEEP Levels on Alveolar Damage and Inflammation in a Model of Open Abdominal Surgery: A Randomized Study in Obese Versus Non-obese Rats
Source: Front Physiol. 2019 Dec 17;10:1513. doi: 10.3389/fphys.2019.01513 (PMC6930834; doi:10.3389/fphys.2019.01513)
Supplement: Supplementary file 2 [file Data_Sheet_2.docx]

**Supplemental Digital Content 2**

**Characterization of obesity**

In adulthood, Obese (Ob) animals had greater body weight **(Figure S2)** and visceral fat mass **(Figure S3)** compared to non-obese (nonOb) animals. In addition, Ob animals had lower lung density, smaller normally aerated lung areas, and larger hypoaerated lung areas compared to nonOb (**Figure S4**).


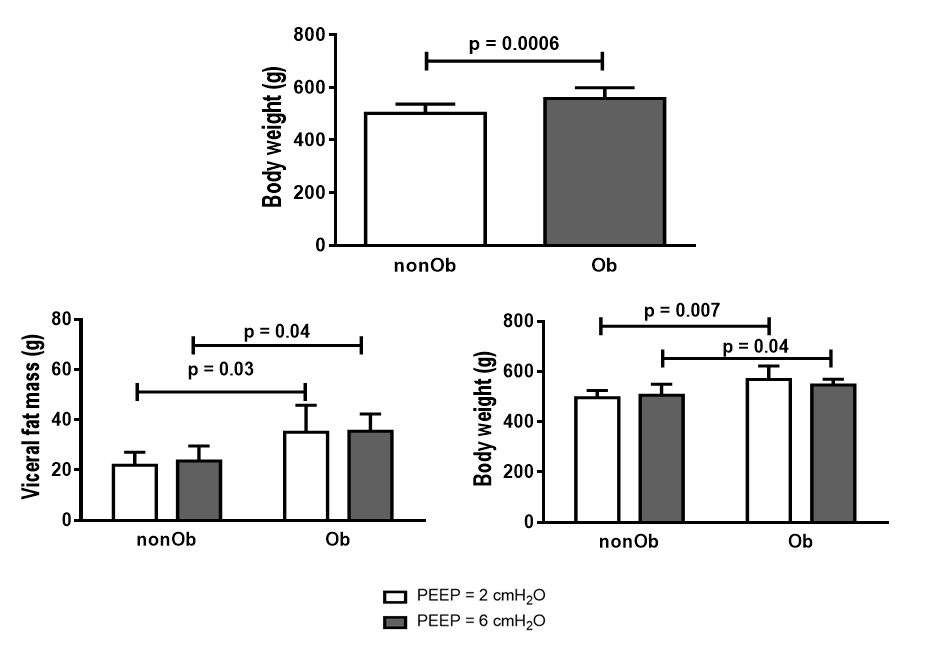


**Figure S2:** Upper panel: Body weight of non-obese and obese rats at 150 days old (n=14/group). Lower panel: Body weight of non-obese and obese rats after randomization and visceral fat mass of non-obese and obese rats after mechanical ventilation (n=7/group). Values are means + standard deviation.


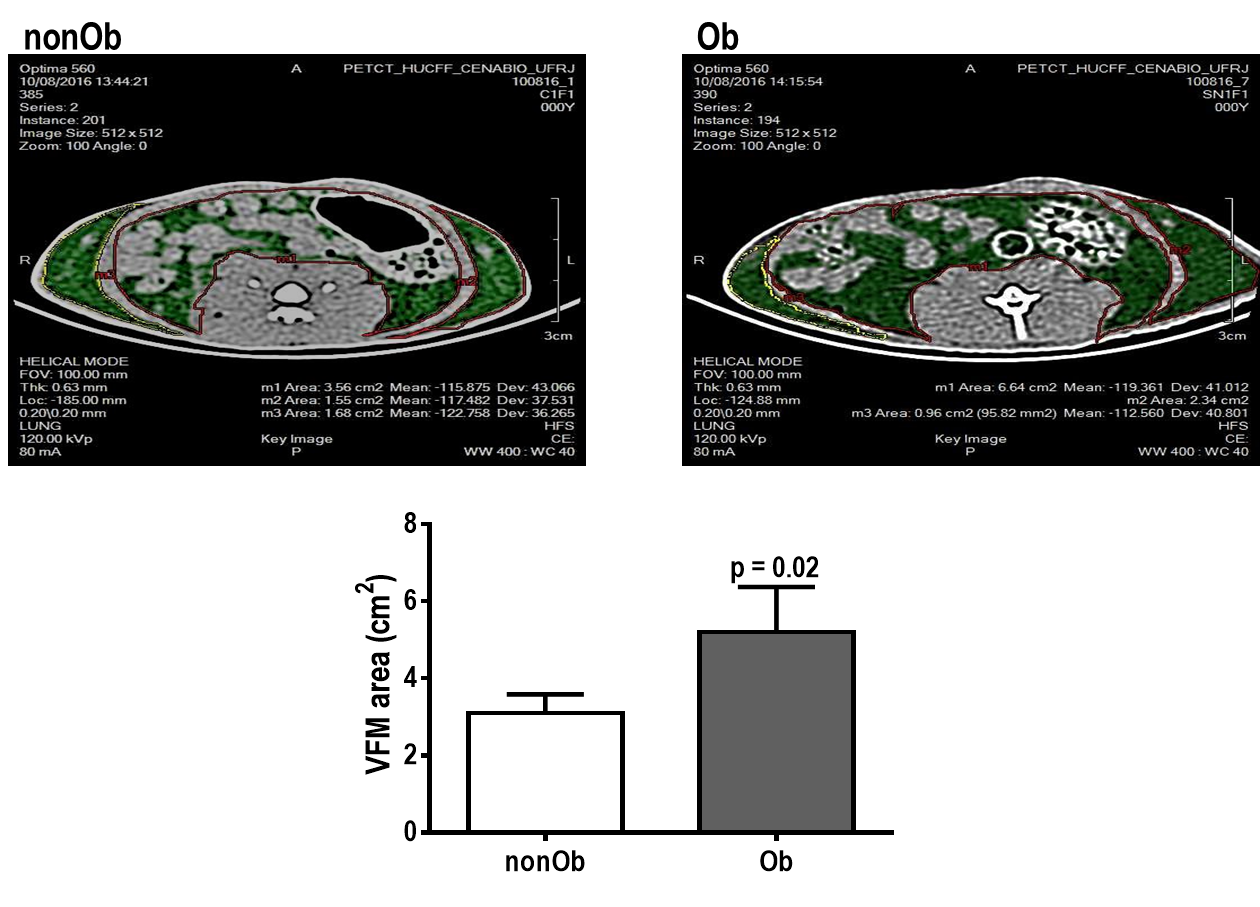


**Figure S3:** Visceral fat mass (VFM) area analyzed by computed tomography (CT) in non-obese (nonOb) and obese (Ob) rats at 150 days old (n=14/group). Values are mean + standard deviation.


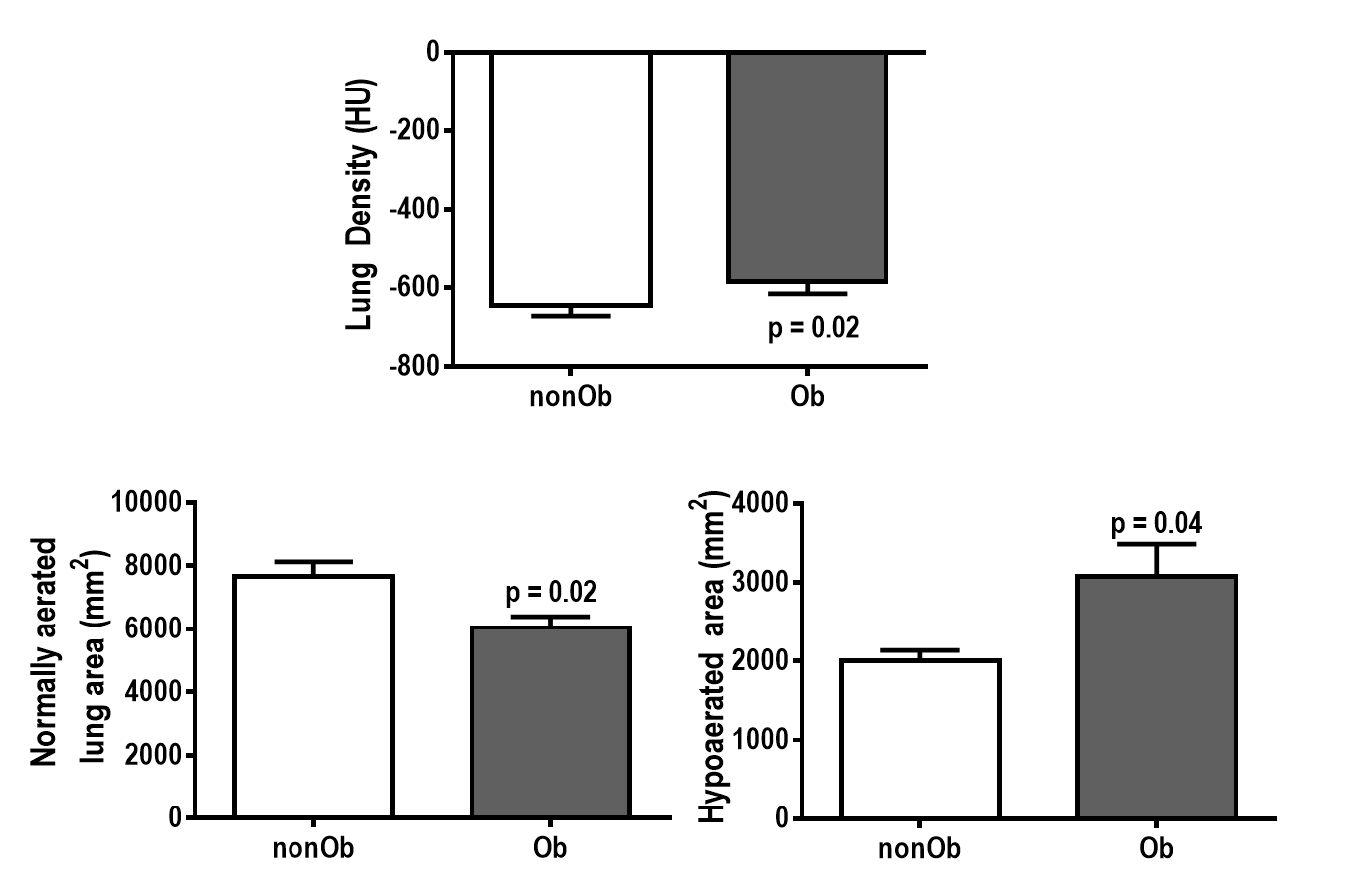


**Figure S4:** Quantitative analysis of computed tomography (CT) analysis of lung parameters. Upper panel: Lung density (HU). Lower panels: Normally aerated and hypoaerated lung areas (mm^2^) of non-obese (nonOb) and obese (Ob) rats at 150 days old (n=14/group). Values are mean ± standard deviation.

**Respiratory parameters and blood gas analysis at Baseline**

At Baseline, respiratory parameters and arterial blood gases were not significantly different between the nonOb and Ob groups, except for mean arterial pressure, which was higher in Ob than in nonOb animals (**Table S2**).

**Table S2:** Respiratory parameters and mean arterial pressure at Baseline

|  | **nonOb** | | **Ob** | |
| --- | --- | --- | --- | --- |
|  | **PEEP2** | **PEEP6** | **PEEP2** | **PEEP6** |
| **V_T_ (mL/kg)** | 6.9 ± 0.4 | 6.8 ± 0.6 | 7.0 ± 0.1 | 6.7 ± 0.9 |
| **RR (bpm)** | 47 ± 2 | 46 ± 2 | 40 ± 3 | 44 ± 5 |
| **Est,L (cmH_2_O/mL)** | 2.0 ± 0.4 | 1.8 ± 0.3 | 3.5 ± 0.3* | 3.6 ± 0.5†* |
| **Est,_W_ (cmH_2_O/mL)** | 0.4 ± 0.2 | 0.3 ± 0.1 | 0.4 ± 0.2 | 0.4 ± 0.2 |
| **pHa** | 7.37 ± 0.02 | 7.37 ± 0.04 | 7.38 ± 0.05 | 7.37 ± 0.04 |
| **PaCO_2_ (mmHg)** | 40 ± 4 | 39 ± 2 | 39 ± 3 | 41 ± 3 |
| **PaO_2_/FiO_2_ (mmHg)** | 360 ± 47 | 394 ± 37 | 267 ± 45* | 279 ± 41† |
| **MAP (mmHg)** | 131 ± 24 | 147 ± 15 | 164 ± 10* | 171 ± 14† |

Values are mean ± SD of seven rats in each group. nonOb/PEEP2: V_T_ = 6 mL/kg with PEEP = 2 cmH_2_O; nonOb/PEEP6: V_T_ = 6 mL/kg with PEEP = 6 cmH_2_O; Ob/PEEP2: V_T_ = 6 mL/kg with PEEP = 2 cmH_2_O; Ob/PEEP6: V_T_ = 6 mL/kg with PEEP = 6 cmH_2_O. At Baseline, all animals were paralyzed and mechanically ventilated with V_T_ = 7 mL/kg, RR to maintain normocapnia (PaCO_2_ 35–45 mmHg; around 45 bpm), inspiratory-to-expiratory ratio = 1:2, fraction of inspired oxygen = 0.4, and PEEP = 2 cmH_2_O for 5 min. V_T_, tidal volume; RR, respiratory rate; Est,L, lung static elastance; Est,w, chest wall static elastance; pHa, arterial pH; PaCO_2_, arterial carbon dioxide partial pressure; PaO_2_/FiO_2_, arterial oxygen partial pressure divided by fraction of inspired oxygen; MAP, mean arterial pressure. *Significantly different from nonOb/PEEP2; †Significantly different from nonOb/PEEP6.


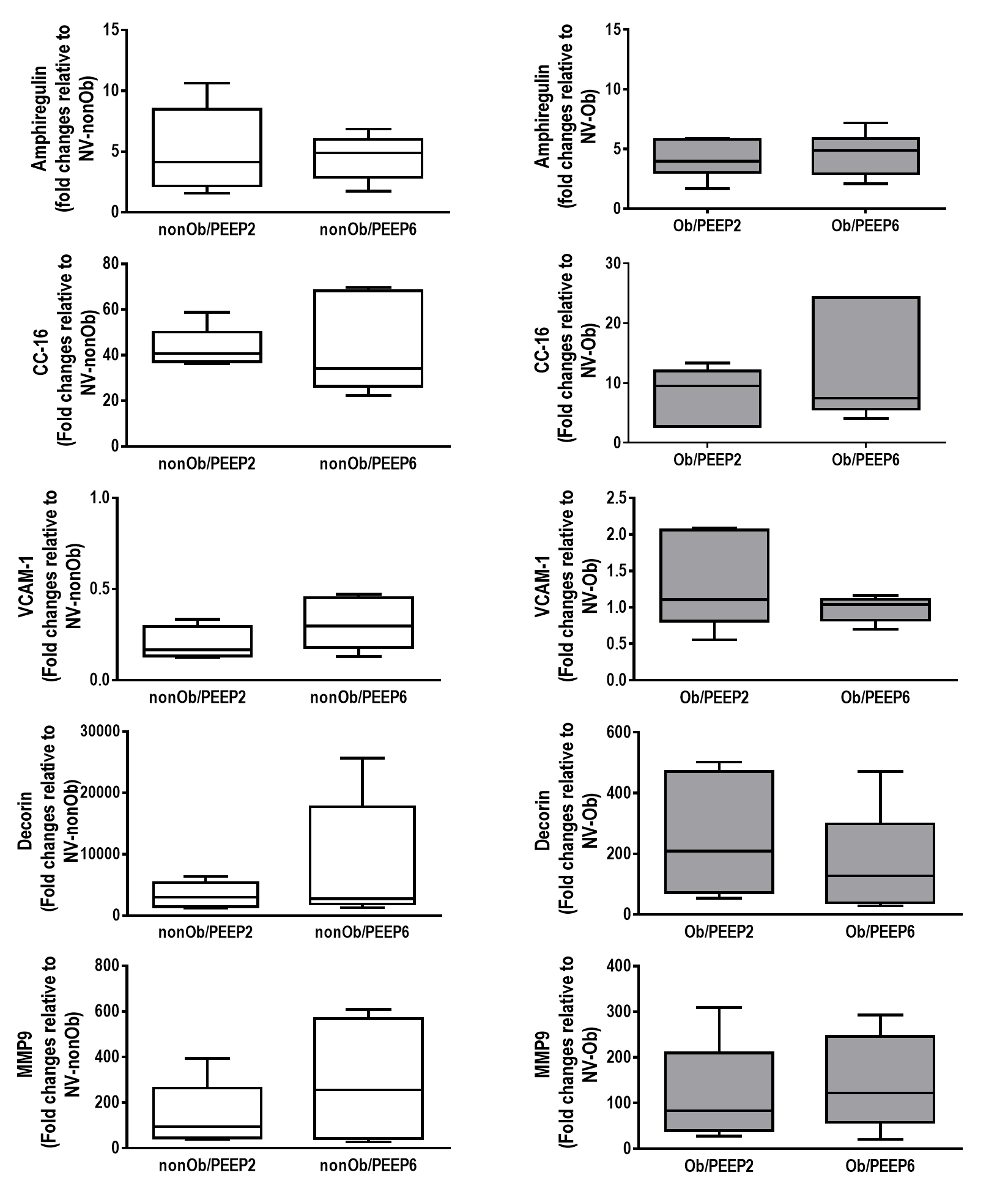


**Figure S5:** Expression of biologic markers associated with lung damage in mechanically ventilated non-obese (nonOb) and obese (Ob) groups. Low PEEP (2 cmH_2_O) and High PEEP (6 cmH_2_O). Real-time polymerase chain reaction analysis of amphiregulin, club cell protein (CC-16), vascular cell adhesion molecule (VCAM)-1, decorin, and metalloproteinase (MMP)-9. Relative gene expression was calculated as a ratio of average expression of each gene to the reference gene (*36B4*) and expressed as fold change relative to non-ventilated animals (NV). Boxes show the interquartile range (25th-75th percentile), while whiskers encompass the range (minimum-maximum) and horizontal lines represent the median in 7 animals/group.
